# Effects of Sodium Bicarbonate Supplementation on Performance and Gastrointestinal Symptoms During a High-Intensity Training Session in Elite Rugby Players: A Double-Blind Randomized Controlled Trial

**DOI:** 10.3390/sports14030100

**Published:** 2026-03-04

**Authors:** Blanca Couce, Selene Baos, Adrián Moreno-Villanueva, Anel E. Recarey-Rodríguez, Juan Mielgo-Ayuso, María Martínez-Ferrán

**Affiliations:** 1 Facultad de Ciencias de la Salud, Universidad Isabel I, 09003 Burgos, Spain; blanca.couce@ui1.es (B.C.); selene.baos@ui1.es (S.B.); adrian.moreno@ui1.es (A.M.-V.); 2 Vicerrectorado de Transferencia, Universidad Internacional de La Rioja, 28224 Madrid, Spain; aneleduardo.recarey@unir.net; 3 Advanced Research in Integrative Physiology for Life Research Group (IAFIV), Facultad de Ciencias de la Salud, Universidad de Burgos, 09001 Burgos, Spain; jfmielgo@ubu.es

**Keywords:** rugby, intermittent exercise, performance, ergogenic aids, gastrointestinal distress, bicarbonate, capillary lactate

## Abstract

Background: Sodium bicarbonate (SB) supplementation can enhance performance in short, high-intensity movements. However, its effectiveness in team sports such as rugby remains insufficiently explored. Methods: In this double-blind, parallel, controlled trial, 17 male professional rugby players ingested SB (0.3 g/kg) or a placebo 90 min before a high-intensity, rugby-specific training session monitored via GPS. The training session was conducted under real-world conditions to enhance ecological validity. Physical performance (countermovement jump, CMJ), fatigue markers (capillary lactate and ratings of perceived exertion, RPE), and gastrointestinal (GI) symptoms were assessed pre- and post-exercise. Results: No significant pre–post changes were observed in CMJ performance in either group. Lactate concentrations increased from pre- to post-exercise in both groups (both *p* < 0.001). The SB group showed higher GI symptom severity before, during and after exercise versus placebo, with several symptoms increasing over time solely in the SB group (*p* < 0.05). RPE increased similarly in both groups (SB: *p* = 0.012; PLA: *p* = 0.008). Due to the small sample size, only moderate-to-large within-group effects and very large between-group differences could be detected; therefore, the study was powered to detect moderate-to-large within-group effects but underpowered for detecting between-group differences. Conclusions: Acute SB ingestion at 0.3 g/kg did not result in detectable improvements in performance or fatigue markers during rugby-specific high-intensity training and was associated with a greater incidence of GI discomfort; however, the study was underpowered to detect small between-group differences. This study was registered on 23 May 2025 on ClinicalTrials.gov (NCT07017582).

## 1. Introduction

During high-intensity, short-duration (<10 min) cyclical exercises, glycolytic metabolism (lactic anaerobic effort) predominates as a primary energy pathway, resulting in significant acid–base disturbances characterized by intramuscular and blood acidification, accompanied by marked hyperlactatemia [1]. This decline in blood pH is due to the accumulation of hydrogen ions (H^+^), decreasing the contractile function in skeletal muscle and, therefore, increasing muscular fatigue [2,3,4,5]. In this context, sodium bicarbonate (SB) is one of the most popular exogenous buffering agents that has been thoroughly studied and has been classified by the Australian Institute of Sports and the International Olympic Committee as a supplement that directly improves sports performance [6,7]. SB supplementation leads to blood alkalosis since its ionized form, bicarbonate (HCO_3_^−^), has the capacity to accept H^+^, as does the endogenous buffer system. Due to the rise in blood pH and HCO_3_^−^ concentration, the transmembrane H^+^ gradient increases. This stimulates the activity of lactate/H^+^ cotransporters, and other transporting systems like monocarboxylate transport proteins, resulting in an enhanced outflow of H^+^ and lactate from the skeletal muscle into the bloodstream, where these metabolites are subsequently buffered or absorbed by nearby or inactive muscle fibers [3,5,6]. Since SB ingestion induces this increased efflux of lactate into the plasma, capillary lactate concentrations may, to some extent, reflect underlying metabolic capacity [8].

SB has been shown to have potentially beneficial effects on high-intensity, short-duration exercise. SB supplementation has shown positive effects from 1 min of maximal effort, with decreasing effectiveness after 10 min [3,5,6]. In terms of performance improvement, increases ranging from 1.7% [3,4] in maximum efforts lasting 1 min after ingesting a dose of 0.3 g/kg of body weight before exercise, to 8% [3,9] in studies involving repeated bouts of supramaximal exercise, have been observed. Nevertheless, it is difficult to accurately quantify an improvement in performance, as studies use different methodologies, dosages, and types and intensities of exercise. In addition, individual variability, training status, and gastrointestinal (GI) distress are also factors that influence the effectiveness of SB supplementation.

There is ample evidence supporting its positive effect on fatigue reduction in high-intensity, very-short-duration sports (0.5–1.5 min) such as 400 m running, 100 m swimming, 500 m rowing or 1000 m speed skating and also in short-duration sports (5–10 min) like 4000 m cycling, 2000 m rowing or 400–800 m swimming [4,6]. Additionally, SB supplement has shown positive effects in endurance sports exceeding 10 min which require a final sprint, such as a 3 h cycling race with a 1.5 min final sprint or 5000 m running [10]. It has also been reported to improve performance in the Yo-Yo test and Wingate test, which are commonly used to assess intermittent sports performance [11,12]. Therefore, SB supplementation may also be advantageous in intermittent high-intensity sports, such as team sports. Although trials have been conducted with SB in basketball, hockey, rugby and soccer players, using specific exercise performance tests, no conclusive results have been obtained regarding its usefulness [12]. There is little evidence to justify its use in team sports whose activity profile does not exactly match the conditions described above [13]. Further research is warranted to examine whether SB supplementation enhances skill performance during team sport-specific exercise, such as in the case of rugby [12]. Rugby union (RU) is an 80 min team sport characterized by numerous repeated bouts of high-intensity, short-duration exercises, such as sprints, mauls and tackles, interspersed with periods of low intensity that serve as recovery, such as standing or walking. In addition, it is necessary to conduct ecological studies in the athletes’ real environment, respecting environmental and training conditions, thus distinguishing them from controlled laboratory conditions, which may lead to different results.

The focus of this study is to explore the ergogenic efficacy of SB and its interaction with gastrointestinal tolerance. Specifically, this study aimed to investigate the effects of acute SB supplementation on physical performance and level of fatigue in professional male rugby players during high-intensity, rugby-specific training, by assessing changes in capillary lactate levels, countermovement jump (CMJ) performance, RPE and GI symptoms.

CMJ and ratings of perceived exertion (RPE) have been widely used in team sports [14,15,16], including rugby [17]. CMJ is a useful objective measure for assessing neuromuscular status as an indicator of fatigue and, consequently, decreased performance, as jump height is correlated with explosive muscle strength and key performance components like power, agility, and speed [18]. RPE has been chosen as a subjective measure of internal training load using a 10-point Borg scale [17]. It was hypothesized that acute SB supplementation recommended by the Australian Institute of Sport [7] (0.3 g/kg, 90 min before exercise) in high-intensity rugby-specific training would (1) improve high-intensity performance outcomes, as evidenced by a statistically significant increase in countermovement jump (CMJ) performance measured pre- and post-training and higher post-exercise lactate concentrations (mmol/L) compared to a placebo condition; (2) reduce RPE, demonstrated by significantly lower session and/or exercise-specific RPE (10-point Borg scale) during and immediately following the training protocol compared with placebo; and (3) be associated with an increase in gastrointestinal symptom incidence without compromising tolerability, indicated by a greater frequency and severity score of self-reported gastrointestinal symptoms such as nausea, bloating, cramping and diarrhea compared to a placebo.

## 2. Materials and Methods

### 2.1. Study Design

This study was a double-blind, parallel-group randomized controlled trial (RCT) registered at ClinicalTrials.gov (NCT07017582) and carried out on a rugby team in the city of Burgos, Spain. The time of submission was 23 May 2025, and it was posted on 12 June 2025. This study was registered retrospectively due to administrative reasons related to the Protocol Registration and Results System (PRS) organization account. Therefore, the registration was done after data collection and analysis were completed, following results that supported publication and further continuation of the research.

Environmental conditions, including ambient temperature (12 °C), relative humidity (71%), and wind speed (22 km/h), were recorded during the training sessions.

### 2.2. Participants

Twenty-two male rugby players who were competing in the First Spanish National League (División de Honor A) initially agreed to participate after attending an informative session on the study where the researchers detailed the characteristics of the study. Regarding inclusion criteria, players had to be free of injury or illness on the test day and agree to temporarily suspend the supplements they were taking for one week prior to the trial. This washout period was done to isolate the actual effect of SB, to avoid carryover effects, and to prevent interactions between supplements. A previous history of bicarbonate intolerance was considered an exclusion criterion. Five players were excluded: three due to injury and two due to illness. No players were excluded on grounds of intolerance because none had previously tried SB supplementation. Thus, seventeen (n = 17; 25.41 ± 5.01 years) players were finally included in the study. Consent to participate in the study was obtained from all rugby players after they were informed verbally and in writing about the protocol. The study was conducted in accordance with the Declaration of Helsinki and approved by the Ethics Committee of Universidad Isabel I (CEI-23-03—PI084). The study was conducted on 14 March 2024, during the competition phase. All athletes trained 4 days a week, with 1 h of physical training in the gym and 1.5–2 h on the field with coaches, plus a fifth match day.

To achieve positional balance across experimental conditions, stratified randomization was performed based on playing position (forwards, n1 = 9; backs, n2 = 8). Participants were subsequently randomized within each stratum to either the intervention group (n1 = 8: forwards; n = 4, backs, n = 5) or the placebo group (n2 = 9: forwards, n = 5; backs, n = 4), ensuring comparable positional representation between groups. Baseline performance was not used in stratification. A researcher from the group that was external to this study was responsible for randomization using alphanumeric codes with Microsoft Excel (version 365, Redmond, WA, USA). No one else had access to the randomization sequence until the trial was completed and the statistical analysis was finalized. Therefore, the researcher responsible for the statistical analysis was also blinded. All players who withdrew were excluded the day before drink administration. Therefore, all randomized players participated in the trial and were measured. Baseline analyses confirmed that there were no significant differences between groups in body composition, age, or any of the variables assessed (Table 1). Therefore, subsequent statistical analyses were conducted by pooling forwards and backs within their respective intervention and placebo groups.

### 2.3. Experimental Design and Procedures

Measurements were taken at four different times: baseline, before training, immediately after training and 1 h after training. The primary outcome was physical performance, which was assessed by CMJ. Secondary outcomes were lactate levels, RPE and the severity of GI symptoms. The training load was monitored via GPS as a descriptive variable in order to characterize the external stimulus.

**Figure 1:**
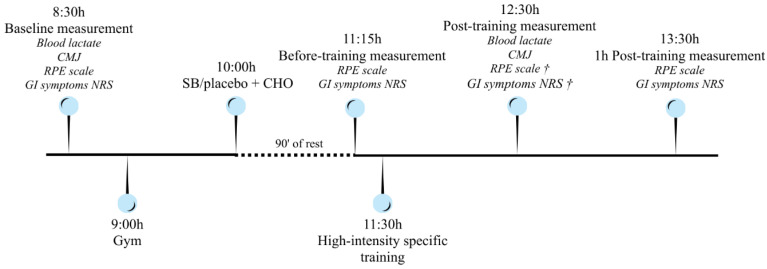
Timeline and structure of the experimental protocol. † The RPE scale and GI symptoms were measured post-training but referred to symptoms perceived during training. RPE and GI symptoms after training were recorded again 1 h after training. NRS (numeric rating scale).

Lactate levels and performance using CMJ were measured at baseline and after exercise, within 1–2 min of completion of the exercise. RPE and GI symptoms were measured on all four occasions. Participants ingested SB supplementation or placebo after the baseline measurement and 90 min prior to initiating the exercise protocol (Figure 1).

Players were monitored throughout the training session via GPS, using 10 Hz multi-constellation GNSS units (Catapult Vector Core X7, Melbourne, Australia), with data processed using the manufacturer’s proprietary software (OpenField, version 2.8.0). Previous studies have reported excellent reliability for Catapult vector 7 devices (ICC = 0.99; CV = 0.17–4.5%) in team sports training sessions [19,20]. The exercise protocol consisted of high-intensity rugby-specific training similar to a real match and all the players were familiar with it. The training session was not standardized to reflect real-world conditions and enhance ecological validity; therefore, GPS metrics were collected for descriptive purposes only and were not used to control or manipulate external load during the training session. Blood lactate levels were measured in capillary samples obtained from the ear lobe using a portable analyzer (Lactate Scout Sport, EKF-diagnostic, Barleben, Germany) whose declared CV ranged from 3 to 8% [21]. To obtain the sample, the ear lobe was pricked with a sterile disposable lancet after being disinfected with an alcohol swab, following the manufacturer’s instructions for use [22,23]. The lancet was medium-flow with a gauge of 0.6 mm and a penetration depth of 1.6 mm (Unisktik 3, Owen Mumford, Woodstock, UK). Performance was evaluated using the CMJ, which was measured using an infrared platform (Optojump, Microgate, Bolzano, Italy). CMJs were performed using established methods [14,17,18]. Starting from an upright position with their hands on their waists, participants performed the jump by flexing their knees to 90° and jumping as high as possible. During the flight stage, they had to extend their knees to 180°, without hyperextending their hips [18]. Two tests were performed at a time with a 10 s standing rest interval between each jump trial [17]. Jump height (cm) was derived from the flight time. The reliability of CMJ variables was assessed using intraclass correlation coefficient (ICC) and coefficient of variation (CV). An ICC (3,1) model was used because all CMJ measurements were obtained using the same device and operator and the aim was to assess test–retest consistency. CMJ performance showed excellent reliability both in pre- and post-measurements (PRE: ICC = 0.96, CV = 2.19%; POST: ICC = 0.96, CV = 2.95%); results were consistent with those obtained in previous studies [24,25].

RPE was evaluated using a 10-point Borg scale [26] adapted by Foster et al. [27]. The questionnaire of GI symptoms included 9 items: nausea, vomiting, stomach pain, flatulence, belching, cramps, urge to defecate, diarrhea and abdominal distension [27,28]. This questionnaire has been previously used in athletic populations, including studies in highly trained and elite athletes [29,30,31], to assess GI responses to SB ingestion, originally adapted from [32]. A numeric rating scale (NRS) between 0 and 10 was used to rate the intensity of the symptoms, where 0 was the absence of symptoms and 10 was the most severe GI distress that can be endured.

### 2.4. Supplementation Protocol

Regarding the supplementation protocol, the intervention group was supplemented with SB (0.3 g/kg body mass), and the placebo group was administered NaCl (0.045 g/kg body mass). NaCl was selected as the placebo at that dose to replicate the salty taste profile of SB, thereby ensuring comparable sensory characteristics between solutions and maintaining participant blinding, consistent with its use in previous SB supplementation studies [9,12,33]. In addition, although sodium intake differed between conditions (0.082 g/kg SB group; 0.017 g/kg placebo group), this placebo provided a partial sodium load to control potential osmotic effects. The amount of liquid was calculated based on body mass (10 mL/kg). However, due to the size of commercial bottles, it was standardized into two groups: 750 mL and 1000 mL bottles. In cases where the calculated volume was less than or equal to 875 mL, the 750 mL bottle was assigned, while if the volume exceeded 875 mL, the 1000 mL bottle was assigned. All bottles were blue plastic to prevent the participants seeing the turbidity of the solution inside. To maintain blinding, the same researcher who performed the randomization prepared the solutions and labeled them. The athletes were provided with a banana and two carbohydrate bars with a bottle of SB or NaCl, and they were asked to drink the bottle within a 15 min period, 90 min prior to the exercise protocol. Although the short intake time may increase GI symptoms, this time restriction was determined to comply with the 90 min between finishing the drink and starting training [7]; thus, this method of administration constitutes a limitation. The trial researchers were present during the consumption of the beverages and subsequently collected all empty bottles. During the rugby-specific training, players were allowed to drink water ad libitum. At the end of the training, participants were asked verbally if they knew which group they belonged to (SB or placebo). Some of the participants who had experienced GI symptoms revealed to suspect that they were in the intervention group.

From the day before until the trial, the participants were asked to avoid all sources of caffeine and alcohol and to have a standardized dinner and breakfast in terms of energy and macronutrients. The participants arrived in the morning after having breakfast at least 3 h before starting the exercise protocol. To maintain optimal hydration levels, participants were asked to maintain their urine color between 1 and 3 using the Armstrong Urine Color Chart [34].

### 2.5. Statistical Analysis

Data are presented as the mean value (standard deviation). Power calculations were performed a priori using G*Power (3.1.9.7) based on the expected change in CMJ performance, which was defined as the primary outcome of the study, given its role as the main indicator of high-intensity performance. With the final allocation (intervention n1 = 8; placebo n2 = 9; total n = 17) and α = 0.05, the detectable standardized effect for paired (within-subject) comparisons across two time points is dz ≈ 0.72 for 80% power. In between-group comparisons of change (n1 = 8, n2 = 9), 80% power corresponds to Cohen’s d ≈ 1.46. Consequently, the study is powered to detect moderate–large changes within-subject but only very large differences in between-group or group × time interactions; smaller effects may remain undetected.

All randomized participants completed the study and were included in the analyses. Data distribution was assessed using the Shapiro–Wilk test.

Between-group differences for normally distributed continuous variables were analyzed using Student’s *t*-test, with effect size (ES) reported as Cohen’s d and interpreted according to Hopkins’ scale [35]: trivial (0–0.2), small (0.2–0.6), moderate (0.6–1.2), large (1.2–2), and very large (>2). When normality assumptions were violated, non-parametric tests were applied. Between-group comparisons were performed using Welch’s *t*-test or the Mann–Whitney U test, depending on variance homogeneity. ESs for the Mann–Whitney U test were reported as rank-biserial (r), interpreted as small (≥0.1), medium (≥0.3) or large (≥0.5) [36].

Changes between groups over time were examined using ANCOVA, including baseline values as covariates. The assumption of homogeneity of regression slopes was tested by including group × baseline interaction terms in the models. When this assumption was violated (significant interaction, *p* < 0.05), between-group differences in change scores (post-exercise minus baseline) were analyzed using independent samples *t*-tests. Effect sizes for ANCOVA were reported as partial eta squared (η^2^), interpreted as small (0.01–0.06), medium (0.06–0.14), or large (≥0.14) [36]. For independent *t*-tests on change scores, effect sizes were reported as Cohen’s d.

Intra-group differences over time were calculated using paired *t*-tests with Cohen’s d as ES, and when normality assumptions were not met, the Wilcoxon test was applied with ES reported as rank-biserial.

Within-group differences across the three time points for ordinal variables were assessed using the Friedman test and Kendall’s W as effect size: small (≥0.1), medium (≥0.3), large (≥0.5), and larger effect (>1) [36]. Given the three comparison time points, pairwise post hoc comparisons were conducted using Conover tests with Holm correction for multiple comparisons.

All analyses were performed using JASP Team (2024) (version 0.18.3, Amsterdam, The Netherlands). Statistical significance was set at *p* < 0.05.

## 3. Results

The GPS monitoring data of the players during training is shown in Table 2. No significant differences were observed between the SB and placebo groups for the GPS-derived variables; only the SB group showed a significantly higher player load compared to the placebo group (*p* = 0.020), showing a moderate effect size (Cohen’s d = 0.570). Although deceleration efforts and total distance showed *p*-values close to the significance threshold (*p* = 0.061 and *p* = 0.063, respectively), these did not reach statistical significance.

The group × time changes in lactate levels and CMJ are shown in Table 3. No significant changes were observed. For lactate, the assumption of homogeneity of regression slopes was violated (significant group × baseline interaction, *p* = 0.018). Consequently, between-group comparisons were conducted on change scores (post − baseline) using independent *t*-tests.

Considering the intra-group changes in lactate levels, both groups showed a significant increase from pre- to immediately post-exercise with a very large effect size (SB group: *p* < 0.001, Cohen’s d = −3.235; placebo group: *p* < 0.001, Cohen’s d = −2.674), where the negative sign indicates higher post-exercise values. In the CMJ, there were no significant differences between the pre- and post-exercise values in either group (SB group: *p* = 0.564; placebo group: *p* = 0.192).

With respect to RPE, no significant differences were observed between groups at baseline (*p* = 1.00), before exercise (*p* = 0.208), during exercise (*p* = 0.355) or after exercise (*p* = 0.159) (Table 4). When changes in RPE were analyzed for each group individually, a similar significant and large change was detected in both groups (SB group: *p* = 0.015; Kendall’s W = 0.693; placebo group: *p* = 0.008, Kendall’s W = 0.796). The post hoc analysis revealed significant differences for both groups from baseline to during and after exercise, and from before exercise to during and after exercise (Table 5).

When analyzing the differences in GI symptoms between groups, the SB group showed significantly higher GI symptom scores than the placebo group, with medium to large effect sizes, before, during and after exercise. Before exercise, the SB group reported higher levels of stomachache (*p* = 0.035, r = −0.690), flatulence (*p* = 0.006, r = −0.929), cramps (*p* = 0.010, r = −0.833) and the urge to defecate (*p* = 0.003, r = −0.976). During exercise, the SB group reported significantly higher scores for flatulence (*p* = 0.013, r = −0.771), belching (*p* = 0.023, r = −0.708), cramps (*p* = 0.042, r = −0.583) and the urge to defecate (*p* = 0.003, r = −0.976). After exercise, belching remained higher in the SB group (*p* = 0.022, r = −0.643). Overall GI symptoms were significantly higher in the SB group before exercise (*p* = 0.003, r = −1.000), during exercise (*p* = 0.005, r = −0.917) and after exercise (*p* = 0.031, r = −0.661).

Analysis of intra-group differences over time regarding GI symptoms showed that the placebo group experienced no changes, whereas the SB-supplemented group suffered increases in belching (*p* = 0.007; Kendall’s W = 0.809) and cramps (*p* = 0.010; Kendall’s W = 0.763) with large ES, as well as increases in overall GI symptoms with moderate ES (*p* < 0.05; Kendall’s W = 0.426). The post hoc pairwise comparisons can be seen in Table 5.

In summary, under the experimental conditions, ingesting 0.3 g/kg of SB prior to rugby-specific training did not result in superior CMJ performance or a distinct blood lactate profile when compared to taking a placebo. RPE increased similarly in both groups over time. However, the SB group reported higher GI symptom severity than the placebo group before, during and after exercise. This included elevated scores for overall symptoms and for specific symptoms such as flatulence, cramps, belching and the urge to defecate. Furthermore, a significant increase in symptom severity was observed over the course of the protocol within the SB group for belching, cramps and overall GI symptoms.

## 4. Discussion

This RCT aimed to evaluate the effects of acute SB supplementation on physical performance, fatigue, and GI symptoms in competitive rugby players during high-intensity rugby-specific training. Our main results are as follows: (1) SB supplementation at 0.3 g/kg was not associated with statistically significant improvements in CMJ performance, blood lactate responses, or RPE compared to the placebo in the present cohort; and (2) this supplementation was associated with a higher incidence of GI symptoms under the conditions of this study. Given the small sample size, the study was underpowered to detect small between-group differences; thus, the null between-group findings should be interpreted cautiously as an absence of evidence rather than evidence of no effect.

The study’s limited sample size meant that only moderate-to-large within-subject changes and very large between-group effects could be reliably detected. It is therefore possible that smaller ergogenic effects—consistent with those reported in previous studies of SB—were present but remained undetected [28,37,38,39]. Nevertheless, the use of stratified randomization ensured that playing positions were evenly represented across conditions, reducing the risk of positional bias and enhancing the internal validity of the comparisons.

Despite using the standard SB dose (0.3 g/kg) and that it was consumed alongside carbohydrates to reduce the likelihood of side effects, the participants still reported significant GI discomfort. This may have offset any potential performance benefits by impairing players’ ability to execute high-intensity movements [40]. We aimed to evaluate the effects of SB supplementation in a practical context, so athletes were allowed to follow their regular training routines. However, the ecological nature of the study, while valuable for external validity, introduced greater variability in training loads and nutrition compared with tightly controlled laboratory conditions, so this may have introduced uncontrolled factors that could have influenced the results, potentially attenuating the measurable effects seen in other investigations [41].

Most existing studies were conducted under controlled laboratory settings [42,43], which may underestimate the extent of GI distress experienced by athletes in real-world training environments. The results are mixed, so the effect of supplementation with SB in intermittent team sports is inconclusive. Some studies on SB supplementation in players from different team sports have obtained similar findings to ours; for example, Cholewa et al. [44] investigated the effects of SB supplementation on the performance of seven highly trained soccer players with the same dose as was used here (0.3 g/kg) 60 min prior to exercise and water ad libitum. No differences were found in the Yo-Yo test between groups (SB and placebo). They obtained post-exercise lactate values very similar to ours, although ours were slightly lower (8.29 ± 2.43 vs. 7.64 ± 1.75 in SB group and 7.66 ± 2.10 vs. 6.32 ± 2.25 in placebo group). No significant interaction existed between the condition and time in the capillary lactate determination nor for distance or time in the case of the fatigue values measured using the Yo-Yo test. This study points to significantly poorer performance in several players in the intervention group.

It is not known whether this is due to GI symptoms from SB supplementation, as these were not measured. However, GI distress is cited as one of the main reasons for dropping out of the study [44].

Durkalec-Michalski et al. [45], in a crossover, double-blind trial, compared chronic SB supplementation (for 8 days) with acute supplementation, placebo and no supplementation. They supplemented 24 male players belonging to the Field Hockey Polish League with SB, using a lower dose than we used (0.2 g/kg SB), a lower volume of fluid (500 mL of water), but a longer time prior to the exercise’s tests (2 h). In addition, they used tablets instead of solutions, which can influence tolerance. The exercise tests consisted of two Wingate anaerobic tests on a cycle ergometer interspersed with a discipline-specific field performance test. The post-exercise lactate levels obtained in the acute supplementation group were considerably higher than ours (17.2 ± 2.2 vs. 7.64 ± 1.75 in SB group and 15.2 ± 3.0 vs. 6.32 ± 2.25 in placebo group). Unlike chronic supplementation, which improved the results of all tests, acute supplementation showed no effects on the results of the Wingate tests, but increased performance in the specific field test. So, they suggest that, in team sports, it is the duration of the SB supplementation that determines the ergogenic effect of SB [45].

On the other hand, Ansdell et al. [46] conducted a crossover trial comparing acute SB supplementation with placebo, using a higher dose than we used (0.4 g/kg), divided into two administrations in order to avoid GI discomfort (0.2 g/kg dissolved in 500 mL of water, one 90 min and the other 60 min prior to exercise) in 10 male nonprofessional basketball players. They conducted an analysis of neuromuscular fatigue during a sprint test that replicated the demands of a 40 min basketball game. The SB group slowed the onset of fatigue by helping preserve the muscle fibers’ contractile components. Nevertheless, in this study, no blood samples were taken to analyze lactate levels [46]. None of these studies measured GI discomfort, despite highlighting it as an impediment to SB supplementation [45,46]. It should be noted that the participants in most studies [44,46] were not professionals, so their level of training was lower, allowing for greater potential for improvement than in well-trained professional athletes. In such well-trained athletes, it is difficult to see improvements in performance despite having increased lactate levels [33,39]. One possible mechanism may be that highly trained athletes have greater muscle buffering capacity and a higher density of monocarboxylate transporter proteins [41]. Athletes with highly developed buffering capacity are likely to be more prone to fatigue because of mechanisms other than acidosis [33]. It is also possible that the tests, even with specific designs to simulate real conditions in each sport, do not cause sufficient acid–base imbalance to overload the natural buffering capacity of highly trained athletes [33,39,44]. Although these mechanisms have been proposed in previous research, they have not been conclusively demonstrated and therefore remain speculative hypotheses. Future studies should consider combining real-world approaches with partial standardization or detailed monitorization of the training load to better control such variables while preserving ecological relevance.

To the authors’ knowledge, there is very limited evidence regarding the effects of SB supplementation in professional rugby union players under real training conditions. Similar to our findings, Cameron et al. [33] conducted a randomized crossover trial involving 25 New Zealand elite male rugby players, using a comparable protocol, although with differences in fluid volume and intake time (0.3 g/kg SB dissolved in 500 mL of isotonic drink and water ad libitum, administered 65 min before a high-intensity, rugby-specific repeated-sprint test). They reported no improvement in sprint test performance in the intervention group, despite observing elevated blood bicarbonate levels, blood pH, lactate levels and lower RPE compared with the control group. In our case, supplementation was associated with a higher player load with no significant differences in RPE nor blood lactate post-exercise. There was also a greater similarity between baseline and post-exercise CMJ with no significant difference within or between groups. Although this study was also conducted on trained rugby union players and similar conclusions were reached, it should be noted that the lactate levels were very different (19.23 ± 0.85 vs. 7.64 ± 1.75 in SB group and 16.05 ± 0.89 vs. 6.32 ± 2.25 in placebo group), probably due to the sporting stimulus to which they were exposed during the test. Participants also experienced a higher incidence of GI discomfort, which remained even 24 h later, interfering with the recovery process and subsequent training sessions [33]. Consistent with this study, our results suggest that, despite interindividual variability in symptom type and intensity, SB is generally not well-tolerated among the professional male rugby union players in this cohort, likely because of the large absolute dose required when scaled to body mass; however, this has not been directly tested and therefore remains speculative.

Another aspect should be noted: previously, as with other ergogenic aids, it has been observed that not all individuals respond to SB supplementation. SB supplementation appears to enhance high-intensity exercise performance in responders, particularly those without gastrointestinal discomfort, whereas non-responders show limited or no benefit, highlighting substantial inter-individual variability in ergogenic response [47].

This study also presents several methodological limitations. First, the relatively small sample size may have limited the statistical power to detect subtle ergogenic effects of SB supplementation and restricts the generalizability of the findings. Specifically, while the study was adequately powered to detect moderate-to-large within-group changes, it was underpowered to detect small between-group differences; therefore, the non-significant between-group findings should be interpreted as an absence of evidence rather than evidence of no effect. SB was dissolved in a fixed fluid volume (750–1000 mL) rather than scaled relative to body mass (10 mL/kg); therefore, the heaviest players ingested a more concentrated solution, increasing GI risk and altering bicarbonate absorption [33]. Although the standardized breakfast minimized dietary variability and ensured sufficient carbohydrate intake, its dose was not adjusted according to body weight, which could have influenced variable glycogen availability and buffering capacity [48]. Furthermore, even though the SB was consumed 90 min before exercise, as indicated in the supplementation protocol, ingestion of the full bolus within 15 min could have exacerbated GI distress before, during and even after training [31,49]. Another limitation is the absence of direct measurements of blood pH, which prevents confirmation of the magnitude of exercise-induced alkalosis following supplementation [31,33]. Neuromuscular fatigue was assessed solely via CMJ performance, without complementary neuromuscular markers. Additionally, although blood lactate sampling was conducted at standardized time points, slight timing deviations relative to exercise cessation (~1 min) may have influenced peak lactate values, introducing a potential timing bias. Likewise, training was not standardized, which could introduce variability in the results, limiting the ability to detect ergogenic effects. Collectively, these factors may have attenuated the potential ergogenic effects of supplementation. Finally, the trial was retrospectively registered, which may limit transparency; nevertheless, the study protocol, outcomes, and analyses were defined a priori and were not modified after data collection.

Despite these limitations, this study has notable strengths. It was conducted in an elite cohort of rugby players under habitual training conditions, maximizing ecological validity and practical relevance. The stratified randomization, double-blind, placebo-controlled design with stratification by position constitutes a major methodological strength, contributing to reduced bias and enhanced internal validity. Objective measures (CMJ and blood lactate concentration) were paired with detailed GI symptom tracking, providing a multidimensional evaluation of the intervention. It is important to note that the supplementation strategy reflected actual practice, as it respected the training routine schedules, ensuring its direct applicability to team sport environments.

From a practical standpoint, the results indicate that, under the conditions of this study, acute ingestion of SB in solution form may be an unsuitable supplementation strategy for elite male rugby union players, because the GI distress outweighs any potential ergogenic benefit. However, given the limited power of between-group comparisons, smaller performance benefits cannot be excluded. Future research should aim to address the current limitations by recruiting larger samples or using crossover designs to improve statistical power. Direct measurements of blood bicarbonate and pH are required to confirm systemic alkalosis and relate physiological responses to performance. Studies should also investigate individualized pre-exercise nutrition and dosing strategies that tailor both bicarbonate concentration and fluid volume to body size. Promising approaches such as enteric-coated capsules, split-dose protocols, or chronic supplementation [12,50] merit further exploration. Position-specific analyses within rugby could determine whether supplementation effects differ between forwards and backs, given their distinct anthropometric and metabolic profiles [51].

## 5. Conclusions

The acute administration of SB at 0.3 g/kg in elite rugby players did not result in detectable improvements in CMJ, lactate responses, or RPE under ecological training conditions and was associated with increased GI symptoms. Therefore, within this specific population and context, an acute bolus of SB does not appear to be a feasible supplementation strategy for professional rugby players under real training conditions. Given the small sample size, the study was sufficiently powered to detect moderate-to-large within-group effects but underpowered to detect small between-group differences; therefore, the absence of statistically significant between-group effects should be interpreted as an absence of evidence rather than evidence of no effect, and smaller ergogenic effects cannot be ruled out. Given the small sample and the likely heterogeneity in bicarbonate uptake and tolerability, practitioners should be cautious in using an acute bolus approach because of the relatively large absolute dose required when scaled to body weight; alternative dosing strategies (enteric-coated capsules, chronic low-dose protocols) and better individualized fluid/nutrition prescriptions merit further investigation in this population.

Based on the results of the present trial, acute supplementation with 0.3 g/kg of SB does not appear to be an appropriate strategy for rugby union players under the conditions of this study. However, from an operational feasibility perspective, and to provide practical guidance for coaches, athletes and sports nutritionists responsible for supplement administration, the following recommendations are proposed in accordance with the International Society of Sports Nutrition’s position statement [12]: (1) Start with the minimum dose that has demonstrated ergogenic effects (0.2 g/kg) 180 min before exercise and ingest with a high-carbohydrate meal in order to minimize the likelihood and severity of gastrointestinal discomfort. (2) Multi-day supplementation protocols should be followed between 3 and 7 days prior to the test (0.4–0.5 g/kg divided into smaller doses consumed across the day, i.e., 0.1–0.2 g/kg with meals).

## Figures and Tables

**Table 1:** Characteristics of the participants.

	Total (n = 17)	SB (n = 8)	PLA (n = 9)	*p*-Value
Age (years)	25.41 (5.01)	25.5 (5.07)	25.33 (5.27)	0.948 ^a^
Body mass (kg)	94.43 (16.65)	97.20 (21.31)	91.97 (11.95)	0.552 ^b^
Body fat (%)	20.00 (6.55)	21.83 (8.12)	18.37 (4.66)	0.292 ^a^
Lean mass (kg)	72.13 (8.24)	72.73 (10.01)	71.61 (6.89)	0.790 ^a^

The table represents the data, comprising the mean value (standard deviation). Body composition was determined using ^a^ Student’s *t*-test and ^b^ Welch’s *t*-test. Abbreviations: SB, group supplemented with SB; PLA, group supplemented with placebo.

**Table 2:** Comparison of Global Positioning System-derived external load variables between groups.

	Total	SB (n = 8)	PLA (n = 9)	*p*-Value	ES
Acceleration efforts (m/s^2^)	41.18 ± 13.97	37.13 ± 11.67	44.78 ± 15.49	0.273 ^a^	0.503
Deceleration efforts (m/s^2^)	61.12 ± 17.87	69.63 ± 18.77	53.57 ± 13.92	0.061 ^a^	0.538
Total distance (m)	5101.53 ± 694.59	5430.58 ± 443.89	4809.03 ± 766.79	0.063 ^a^	0.538
High-speed distance (m)	679.97 ± 275.18	586.75 ± 172.05	762.83 ± 330.35	0.236 ^b^	0.281
Player load	498.56 ± 91.53	551.18 ± 55.32	451.78 ± 94.06	0.020 ^a^	0.570
Max velocity (km/h)	26.21 ± 2.28	26.73 ± 2.53	25.74 ± 2.08	0.387 ^a^	0.496
Work/rest ratio	0.16 ± 0.03	0.15 ± 0.02	0.16 ± 0.04	0.883 ^b^	0.281

The table represents the data with the mean value ± standard deviation. ^a^ Student’s *t*-test with Cohen’s d as effect size or ^b^ Mann–Whitney U test with effect size reported as rank-biserial. Abbreviations: ES (effect size), PLA (group supplemented with placebo), SB (group supplemented with sodium bicarbonate).

**Table 3:** Within- and between-group comparisons of blood lactate and countermovement jump performance at baseline and post-exercise in sodium bicarbonate group (n = 8) and placebo group (n = 9).

	Group	Baseline	Post-Exercise	Intra-Group × Time Differences ^a,b^		Group × Time Differences ^c,d^	
				*p*-Value	ES	*p*-Value	ES
Lactate (mmol/L)	SB	0.95 ± 0.52	7.64 ± 1.75	<0.001 ^a^	−3.235	0.240 ^c^	−0.594
	PLA	0.86 ± 0.36	6.32 ± 2.25	<0.001 ^a^	−2.674		
CMJ (cm)	SB	39.88 ± 7.05	39.48 ± 7.26	0.564 ^a^	0.214	0.427 ^d^	0.009
	PLA	42.70 ± 4.50	41.62 ± 5.75	0.192 ^b^	0.511		

The table presents the data, comprising the mean value ± standard deviation. Intra-group × time differences: ^a^ paired *t*-test with Cohen’s d as effect size or ^b^ Wilcoxon’s test with effect size reported as rank-biserial. Group × time differences: ^c^ independent *t*-test with Cohen’s d as effect size ^c^ or ^d^ ANCOVA with eta squared (η^2^) as effect size. For lactate, ANCOVA assumptions were violated (significant group × baseline interaction, *p* = 0.018); therefore, between-group comparison was performed on change scores using independent *t*-test. For CMJ, ANCOVA assumptions were met and results are reported accordingly. Abbreviations: countermovement jump (CMJ), ES (effect size), PLA (group supplemented with placebo), SB (group supplemented with SB).

**Table 4:** Comparison of gastrointestinal symptoms and ratings of perceived exertion between sodium bicarbonate and placebo groups at each time point.

Variable	Group	Baseline			Pre-Exercise			During Exercise			Post-Exercise		
			*p*-Value	ES		*p*-Value	ES	*p*-Value	ES			*p*-Value	ES
Nausea	SB	0.00 ± 0.00	-	-	2.14 ± 2.27	0.086 ^b^	−1.062 ^b^	2.50 ± 2.51	0.060	−0.542	1.14 ± 2.04	-	-
	PLA	0.63 ± 1.19			0.33 ± 0.82			0.50 ± 1.41			0.00 ± 0.00		
Vomiting	SB	0.00 ± 0.00	-	-	0.43 ± 0.79	-	-	0.50 ± 1.23	-	-	0.14 ± 0.38	-	-
	PLA	0.13 ± 0.35			0.00 ± 0.00			0.00 ± 0.00			0.00 ± 0.00		
Stomachache	SB	0.00 ± 0.00	-	-	4.86 ± 2.97	**0.035**	**−0.690**	4.33 ± 3.62	-	-	2.43 ± 3.36	-	-
	PLA	0.00 ± 0.00			1.00 ± 2.5			0.00 ± 0.00			0.00 ± 0.00		
Flatulence	SB	2.88 ± 2.48	0.232	−0.359	6.00 ± 2.00	**0.006**	**−0.929**	4.83 ± 2.86	**0.013**	**−0.771**	3.00 ± 2.58	0.059	−0.554
	PLA	1.50 ± 1.69			1.00 ± 1.67			0.63 ± 1.19			0.63 ± 1.41		
Belching	SB	0.38 ± 0.74	0.643	−0.109	4.57 ± 3.91	**0.028** ^b^	**−1.501** ^b^	4.17 ± 3.06	**0.023**	**−0.708**	2.43 ± 2.37	**0.022**	**−0.643**
	PLA	0.25 ± 0.71			0.33 ± 0.82			0.50 ± 0.93			0.25 ± 0.71		
Cramps	SB	1.25 ± 1.83	0.240	−0.281	6.14 ± 3.19	**0.010**	**−0.833**	4.33 ± 3.93	0.063 ^b^	−1.297 ^b^	3.00 ± 3.51	**0.043**	**−0.571**
	PLA	0.25 ± 0.71			0.50 ± 1.23			0.50 ± 1.41			0.75 ± 2.12		
Urge to defecate	SB	2.13 ± 2.53	0.304	−0.281	8.00 ± 2.89	**0.003**	**−0.976**	5.33 ± 4.18	**0.030** ^b^	**−1.696** ^b^	4.71 ± 4.15	0.057	−0.536
	PLA	0.88 ± 1.81			0.33 ± 0.820			0.25 ± 0.71			1.25 ± 3.54		
Diarrhea	SB	1.00 ± 2.14	0.590	−0.125	7.71 ± 3.59	-	-	4.67 ± 5.16	0.107 ^b^	−1.101 ^b^	5.00 ± 5.00	0.093 ^b^	−0.985 ^b^
	PLA	0.38 ± 1.06			0.00 ± 0.00			0.50 ± 1.41			1.00 ± 2.83		
Abdominal distension	SB	0.50 ± 0.93	-	-	3.86 ± 3.43	-	-	2.33 ± 2.88	0.187 ^b^	−0.845 ^b^	1.57 ± 2.64	0.207	−0.321
	PLA	0.00 ± 0.00			0.00 ± 0.00			0.50 ± 1.07			0.25 ± 0.71		
Overall GI symptoms	SB	0.90 ± 0.71	0.170	−0.422	4.86 ± 1.65	**0.003**	**−1.000**	3.67 ± 2.07	**0.005**	**−0.917**	2.60 ± 2.27	**0.031** ^b^	**−0.661** ^b^
	PLA	0.44 ± 0.50			0.399 ± 0.53			0.38 ± 0.57			0.46 ± 1.00		
RPE	SB	2.75 ± 2.05	1.000	0.018	3.29 ± 1.70	0.208	−0.429	7.83 ± 1.17	0.355	−0.313	7.14 ± 1.22	0.159	−0.429
	PLA	2.71 ± 1.98			2.50 ± 1.38			6.75 ± 1.98			5.25 ± 2.49		

The table represents the data, comprising the mean value ± standard deviation. Bold values indicate statistical significance (*p* < 0.05). Inferential statistics could not be calculated for some time points due to the absence of variability, as all participants reported identical values (i.e., zero). Differences between groups were identified using the Mann–Whitney U test with effect size reported as rank-biserial. ^b^ Welch’s *t*-test was used instead of the Mann–Whitney U test where appropriate. Abbreviations: GI (gastrointestinal), PLA (group supplemented with placebo), rate of perceived exertion (RPE), SB (group supplemented with SB).

**Table 5:** Changes within groups in gastrointestinal symptoms and ratings of perceived exertion over time.

	SB (n = 8)			PLA (n = 9)		
Variable	*p*-Value	ES	Post Hoc	*p*-Value	ES	Post Hoc
Nausea	0.106	0.407	-	0.392	0.167	-
Vomiting	0.290	0.250	-	0.392	0.167	-
Stomachache	0.066	0.479	-	0.392	0.167	-
Flatulence	0.118	0.391	-	0.504	0.130	-
Belching	0.007	0.809	B-PRE (<0.001) B-DURING (<0.001) PRE–POST (0.011) DURING–POST (0.011)	0.392		-
Cramps	0.010	0.763	B-PRE (<0.001) B-DURING (0.007) PRE vs. POST (0.014)	0.861	0.042	-
Urge to defecate	0.371	0.209	-	0.392	0.167	-
Diarrhea	0.295	0.247	-	0.392	0.167	-
Abdominal distension	0.056	0.504	-	0.145	0.300	-
Overall GI symptoms	0.002	0.426	B-PRE (<0.001) B-DURING (<0.001) B-POST (<0.005) PRE–DURING (0.005) PRE–POST (<0.001) DURING–POST (0.006)	0.335	0.189	-
RPE	0.015	0.693	B-DURING (0.007) B-POST (0.017) PRE–DURING (0.017) PRE–POST (0.034)	0.008	0.796	B-DURING (<0.001) B-POST (0.018) PRE–DURING (<0001) PRE–POST (0.010)

Friedman’s test with Kendall’s W is given as effect size. Pairwise post hoc comparisons were conducted using Conover’s tests with Holm’s correction for multiple comparisons (only significant pairwise comparisons are shown in the post hoc column). Abbreviations: B (baseline), DURING (during exercise), ES (effect size), GI (gastrointestinal), PLA (group supplemented with placebo), PRE (pre-exercise), POST (post-exercise), RPE (rate of perceived exertion), SB (group supplemented with SB).

## Data Availability

The data presented in this study are available on request from the corresponding author due to privacy regulations.
